# Trends in Method of Diagnosis of Type 2 Diabetes Mellitus: Results from SHIELD

**DOI:** 10.1155/2009/796206

**Published:** 2009-04-14

**Authors:** Helena W. Rodbard, Andrew J. Green, Kathleen M. Fox, Susan Grandy

**Affiliations:** ^1^Endocrine & Metabolic Consultants, Rockville, MD 20852, USA; ^2^Midwestern Endocrinology, Overland Park, KS 66211, USA; ^3^Strategic Healthcare Solutions, LLC, Monkton, MD 21111, USA; ^4^AstraZeneca LP, Wilmington, DE 19803, USA

## Abstract

*Aims*. This study assessed whether recent screening recommendations have led to increased diagnosis of type 2 diabetes mellitus (T2DM) through routine screening. *Methods*. Respondents to the 2006 US SHIELD survey reported whether a physician told them they had T2DM, age at diagnosis, specialty of the physician who made the diagnosis, and whether the diagnosis was made after having symptoms, during routine screening, or when being treated for another health problem. *Results*. Of 3 022 T2DM respondents, 36% of respondents reported that T2DM diagnosis was made during routine screening alone, 20% after having symptoms alone, and 6% when being treated for another health problem alone. The proportion of T2DM respondents reporting a diagnosis based only on screening increased approximately 42% over a 15+-year time span (absolute increase from 31% to 44%) (*P* < .001), whereas symptom-based diagnosis did not change significantly (*P* = .10). T2DM was diagnosed primarily by family physicians (88.3%). *Conclusion*. These findings highlight the importance of regular screening for diabetes and the vital role of primary care physicians in recognizing individuals with T2DM.

## 1. Introduction

An estimated 20.6 million adults in the United States have diabetes mellitus [1]. In patients newly diagnosed with type 2 diabetes, complications are often present at the time of diagnosis, suggesting that clinical onset of the disease occurred years prior to diagnosis [2, 3]. Symptoms of diabetes often go unrecognized or are assumed to be insignificant [4], resulting in a delay of diagnosis.

At present, the American Diabetes Association (ADA) endorses screening of individuals at high risk for diabetes but indicates that there is insufficient evidence to support cost-effective screening of all asymptomatic individuals [5]. The ADA recommends screening be considered at 3-year intervals beginning at age 45, and in adults with body mass index (BMI) ≥ 25 kg/m^2^ and who have additional risk factors (e.g., family history of diabetes, physical inactivity, certain races/ethnicities, hypertension, dyslipidemia). The 2007 American Association of Clinical Endocrinologists (AACE) guidelines recommend annual screening for all individuals 30 years of age and older who are at risk for type 2 diabetes mellitus [6].

In a survey of patients diagnosed with type 2 diabetes in 2003, researchers found that all diagnoses for type 2 diabetes were either serendipitous, in response to specific symptoms, or patient initiated [7]. Clark et al. found that most patients with a diagnosis of diabetes have little symptomatology or that the ADA checklist of symptoms associated with diabetes lacks specificity for the disease [8]. A study of 504 members of a medical plan in Minnesota diagnosed with diabetes between 1995 and 1996 reported that the diagnosis was made for approximately 50% of the patients at the time of routine visits (e.g., preventive care visits, chronic disease visits, preoperative assessments), and that the diagnosis for 43% of patients was made at acute care visits (e.g., visit for acute respiratory infection, pain, cellulitis) [9]. These findings suggest that symptoms may be the initiating factor for a small percentage of healthcare visits, and that there is considerable variability in the care setting in which the diagnosis of diabetes is made.

The Study to Help Improve Early evaluation and management of risk factors Leading to Diabetes (SHIELD) is a longitudinal, observational study of individuals with or at risk for diabetes. This survey provides an opportunity to examine the means by which a large group of individuals with type 2 diabetes mellitus were diagnosed, and to determine whether there have been changes in patterns of diagnosis over time. This study describes how individuals learned they have type 2 diabetes, the specialties of the physicians who made the diagnosis, and the rates of screening for type 2 diabetes. The purpose of the study was to determine if diabetes screening rates have increased in recent years among individuals with type 2 diabetes mellitus.

## 2. Materials and Methods

The SHIELD study has 3 phases extending over 5 years: (1) an initial screening phase to identify cases of interest in the general population; (2) a baseline survey for identified cases to collect health status, health knowledge and attitudes, and current behaviors and treatments; and (3) four annual surveys to follow disease progression in individuals with an established diagnosis of diabetes, and the rate of transition from at risk to a diagnosis of diabetes.

The SHIELD survey methodology has been described in detail previously [8, 10]. Briefly, the screening survey was mailed on April 1, 2004, to a stratified random sample of 200 000 US households representative of the US population for geographic residence, household size and income, and age of head of household, identified by the Taylor Nelson Sofres National Family Opinion (TNS NFO) panel. The screening survey was designed to identify individuals with diabetes and those with cardiometabolic risk factors. A response rate (computed as completed surveys/surveys mailed) of 63.7% was obtained from 127 420 households (containing 211 097 adults).

The baseline survey was mailed in September and October 2004 to a representative sample of individuals, independently sampled (*n* = 22 001), who were identified in the screening survey as having type 1 diabetes, type 2 diabetes, or one of 6 risk factors (abdominal obesity, BMI ≥ 28 kg/m^2^, reported diagnosis of “cholesterol problems,” reported diagnosis of high blood pressure/hypertension, and history of cardiovascular disease). Each respondent group was balanced to be representative of that population for age, gender, geographic region, and household size and income for the US population, and then a random sample from each group was selected and sent the baseline survey. A response rate of 71.8% was obtained (*n* = 15 794).

In August 2005, the first annual follow-up survey was mailed to the baseline survey respondents still enrolled in the TNS NFO panel (*n* = 19 613). The second annual follow-up survey was mailed in July 2006 to individuals who had returned either or both the baseline and first annual questionnaires (*n* = 18 445). A 75% response rate was obtained for the 2006 follow-up survey (*n* = 13 877), with 3 022 individuals with type 2 diabetes mellitus.

### 2.1. Time Since Self-Reported Diagnosis of Type 2 Diabetes Mellitus

Respondents who self-reported a diagnosis of type 2 diabetes mellitus were asked to indicate the age at which they were diagnosed. Time since diagnosis was computed by subtracting age at diagnosis from current age and categorized into 3-year increments to capture changes before and after the ADA screening guidelines published in 2004 [5]. These 3-year increments also coincide with the ADA recommendation for screening at 3-year intervals. This resulted in six categories for time since diagnosis: diagnosis made <3 years, 3 to 5 years, 6 to 8 years, 9 to 11 years, 12 to 14 years, or ≥15 years previously. Age at diagnosis for type 2 diabetes mellitus was >21 years since type 1 diabetes respondents were classified as age at diagnosis of ≤21 years. Individuals between 22 and 45 years of age were included because of the recent increase in diabetes diagnosis at younger ages.

### 2.2. Method of Diagnosis

Respondents who self-reported a diagnosis of type 2 diabetes mellitus were asked whether they were diagnosed because they were tested after having health symptoms (symptoms), during routine screening/lab work (screening), or when being treated for another health problem (other health problem). These categories were used to determine whether the 2004 ADA screening recommendations [5] have led to a trend toward increased diagnoses for type 2 diabetes mellitus as a result of routine screening and decreased diagnoses based on symptoms. Specifically, respondents were asked: “How did you find out that you had diabetes? Was it found… (Check all that apply)” with response categories of “during routine screening/lab work (blood test, etc.) ordered by my doctor”; “when I was tested for it after having some health symptoms”; or “when I was being treated for another health problem.” Respondents could also check that they were diagnosed “with home testing” or “other,” but these responses were considered uninformative for the purposes of this study and not included in the analysis. Respondents were permitted to check multiple responses.

Respondents with type 2 diabetes mellitus were also asked to indicate the specialty of the physician who made their diagnosis (e.g., family doctor/general practitioner, endocrinologist, cardiologist, neurologist, or other specified physician).

In addition, individuals were asked whether they had a cardiovascular event, defined as “heart disease/heart attack, stroke/transient ischemic attack (TIA), or narrow/blocked arteries/carotid artery disease.” Because a cardiovascular event may be a trigger for screening and detecting diabetes mellitus, a subgroup analysis was conducted of individuals who self-reported a cardiovascular event and who were given a diagnosis of type 2 diabetes within a year of the cardiovascular event.

### 2.3. Statistical Analysis

Comparisons of the proportion of individuals reporting a method of diagnosis over time were made using chi-square tests for trends. Two-sided *P* values less than .05 were considered significant.

## 3. Results

There were 3 022 respondents with type 2 diabetes mellitus from the 2006 SHIELD survey, and more than half of these individuals were female (59.3%), white (85.0%), of non-Spanish ethnicity (91.6%), and obese (63.4%) (Table 1). There were 2 749 respondents with type 2 diabetes mellitus who reported only one method of diagnosis, and 273 respondents who selected 2 or more methods of diagnosis.

Respondents with type 2 diabetes mellitus reported that they had had the condition for an average of 10 years (median: 8 years) (Table 1). Approximately 22% of respondents received the diagnosis of type 2 diabetes mellitus ≥15 years previously, compared with 14% of respondents who were diagnosed <3 years previously (Table 1).

### 3.1. How Individuals Received the Diagnosis of Type 2 Diabetes Mellitus

For all respondents (including those who selected multiple methods of diagnosis), 63% of respondents reported a diagnosis of type 2 diabetes mellitus based on screening, 49% reported a diagnosis based on symptoms, and 21% reported a diagnosis based on other health problems. Respondents who selected only one method of diagnosis were then analyzed to assess differences over time without confounding with multiple choices. Analysis of solely the individuals reporting only one method of diagnosis showed that 36% of individuals reported a diagnosis of type 2 diabetes mellitus based on screening alone (Table 2). Twenty percent of individuals reported that the diagnosis was based on symptoms alone, while 6% of individuals reported that the diagnosis was based on another health problem alone (Table 2).

**Figure 1:**
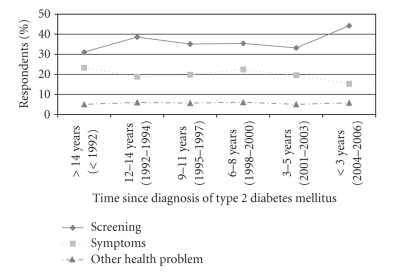
*Method of diagnosis for SHIELD individuals with type 2 diabetes mellitus,* *n* = 2 749*. *Data are shown only for those respondents who selected only one of three categories: routine screening, symptoms, or other health problem. *P* < .001 for routine screening change over time, *P* = .10 for symptoms change over time, *P* = .59 for other health problem change over time.

To determine if there was a trend over time in the method of diagnosis, the proportion of respondents given the diagnosis by each method was computed for each 3-year interval since diagnosis. The percentage of individuals self-reporting the diagnosis of type 2 diabetes mellitus based on screening alone increased approximately 42% over time, from 31% in the ≥15 years category to 44% in the <3 years category (*P* < .001). In contrast, the percentage of individuals receiving a diagnosis based on symptoms alone decreased by 34% and the percentage of individuals diagnosed when being treated for another health problem alone increased 14% over time; these changes were not statistically significant (*P* = .10 for symptoms and *P* = .59 for other health problem) (Figure 1).

There were 273 (9.0%) respondents with type 2 diabetes mellitus who reported more than one method of diagnosis (e.g., they selected both screening and symptoms). Because respondents could select multiple responses, a separate analysis was conducted to determine whether the trend in method of diagnosis differed for respondents who self-reported any combination of responses; however, no differences emerged.

### 3.2. Specialty of Physicians Who Made the Diagnosis of Type 2 Diabetes Mellitus

Most individuals with type 2 diabetes mellitus reported that they received their diagnosis from their family doctor or general practitioner (88.3%) (Table 3). Few reported that an endocrinologist (4.4%) had made the diagnosis, and even fewer reported that a cardiologist (0.5%) or neurologist (0.7%) had made the diagnosis. This pattern did not differ significantly with respect to duration of diabetes.

### 3.3. Cardiovascular Events

There were 826 individuals with type 2 diabetes mellitus who reported a cardiovascular event (myocardial infarction, stroke) and age at time of event. Of these individuals, 141 (17.1%) received the diagnosis of diabetes mellitus within the year following the cardiovascular event, 23% indicated that the diagnosis was made during routine screening, 21% reported that it was based on symptoms, and 13% indicated being diagnosed when being treated for another health problem.

## 4. Discussion

The 2004 ADA guidelines recommend screening of individuals at high risk for diabetes mellitus on a 3-year cycle starting at age 45 [5]. Early diagnosis of type 2 diabetes mellitus is a central goal. The SHIELD findings reveal that in individuals with type 2 diabetes mellitus, the proportion of individuals with type 2 diabetes mellitus detected by screening had increased from 2001–2003 to 2004–2006. Most of the type 2 diabetes mellitus respondents were obese and had hypertension and/or dyslipidemia, making them appropriate candidates for diabetes screening.

These results also indicate that there was no significant change over time in the rate of diagnosis of type 2 diabetes mellitus due to symptomatic presentation or while being treated for other health problems. A previous investigation using SHIELD survey data found that approximately 70% of respondents did not report having experienced the typical symptoms of diabetes from the ADA checklist, for example, frequent urination and increased fatigue [8]. Moreover, only 45% of respondents at high risk for developing diabetes (had 3 or more cardiometabolic risk factors) reported one or more of the classic symptoms of diabetes, and 55% reported no symptoms [8]. The presence of one or more of these classic symptoms alone was generally not sufficient impetus for an individual to consult a physician [8].

The proportion of SHIELD respondents diagnosed on the basis of another health problem was low (<10%). Routine physician visits may be an ideal time and setting to implement screening. A study found that approximately half of the patients were diagnosed at routine or scheduled physician visits, and 43% of patients were diagnosed at acute care visits [9]. In the face of the rapidly increasing incidence of diabetes mellitus, it is important that physicians identify individuals at risk for diabetes mellitus during routine visits and test appropriately. Additionally, other venues for screening should be explored, such as the new diabetes screening program for blood donors at the Blood Bank of Delmarva [11].

A cardiovascular event may serve as a trigger for screening and detection of type 2 diabetes. It was not apparent in this study whether patients who experienced a cardiovascular event were more likely to be screened for type 2 diabetes. Screening was the most frequently mentioned self-reported reason for diagnosis in these individuals. However, we were unable to distinguish those individuals who were diagnosed with type 2 diabetes mellitus specifically because they had a cardiovascular event from those in whom the diagnosis was made for other reasons. We had anticipated that specialists such as cardiologists would have been responsible for an increasing percentage of diagnoses, especially for respondents with a cardiovascular event. However, our results did not confirm this hypothesis. Generalists (family doctors/general practitioners) continue to be the predominant specialty of physician making the diagnosis of type 2 diabetes.

Limitations of this study include the possible bias introduced by having only a small percentage (5%–8%) of households agree to participate in the panel, and the data tend to underrepresent the very wealthy and very poor segments of the population, and do not include military and institutionalized individuals [12, 13]. Respondents who selected home testing or “other” method of diagnosis (38%) instead of screening, symptoms or other health problem for diabetes were not included in the analyses since it could not be determined if a physician had ever diagnosed them. The exclusion of these respondents may affect the generalizability of the study findings. The SHIELD survey relied upon self-reporting of clinical data, including the diagnosis of type 2 diabetes mellitus, method of diagnosis, age at diagnosis, and the specialty of the physician making the diagnosis, without independent confirmation by the physician or examination of medical records. Recall of this type of information by the respondent could potentially differ for recently diagnosed respondents compared with respondents given the diagnosis more than 15 years previously (median years since diagnosis = 8). Prior analyses have demonstrated generally good correlation between prevalence of type 2 diabetes mellitus as assessed as self-report in SHIELD when compared with the prevalence of diabetes mellitus determined by objectively measured survey such as the National Health and Nutrition Examination Survey (NHANES) [10, 14]. Although accuracy and validity of the self-reported method of diagnosis and age at diagnosis were not done in this study, other studies have shown a significant correlation and validity of self-reported data over long recall periods [15–17]. Age at menopause reported at an interval of almost 20 years showed significant correlation with a mean difference between first-reported and recalled menopause of 0.5 years [15]. Breast cancer survivors accurately recalled their treatment and number of invaded nodes with kappa ≥0.85 [16]. Women accurately recalled their cause of subfertility after 5.5 years with kappas of 0.50–0.79 [17]. However, this study does provide data regarding the method of diagnosis of type 2 diabetes mellitus in a large sample of respondents who are representative of the US population with a high survey response rate.

## 5. Conclusions

This study showed that diagnosis of type 2 diabetes mellitus by routine screening has increased significantly over the previous 15 years, with the increase occurring in the most recent years of 2004–2006. However, recognition of typical symptoms of hyperglycemia and medical testing as part of care for acute or other illnesses represent important means of detection. These data support the need for more aggressive screening in order to maximize early detection and the benefits of early intervention.

## Figures and Tables

**Table 1:** Characteristics of SHIELD respondents in 2006 diagnosed with type 2 diabetes mellitus.

Characteristics	Type 2 diabetes mellitus
	*n* = 3 022
Gender, women, %	59.3
Age, years, mean (SD)	61.3 (12.1)

Race	

White, %	85.0
Black, %	9.6
Asian or Pacific Islander, %	0.6

Annual household income, %	

<$20 000	25.4
$20 000–$34 999	20.4
$35 000–$54 999	19.9
$55 000–$84 999	17.4
≥$85 000	16.8

Body mass index (BMI), kg/m^2^	

Underweight or normal weight (BMI ≤ 24.9), %	9.3
Overweight (BMI 25.0–29.9), %	24.9
Obese (BMI ≥ 30), %	63.4

Cardiometabolic risk factors	

Abdominal obesity,^§^ %	83.4
BMI ≥ 28 kg/m^2^, %	77.0
Dyslipidemia diagnosis,^§^ %	82.5
Hypertension diagnosis,^§^ %	75.9
Prior cardiovascular event,^§^ %	41.7

Time since type 2 diabetes mellitus diagnosis, years	

Mean (SD)	10.1 (8.4)
<3 years, %	13.8
3–5 years, %	21.9
6–8 years, %	17.8
9–11 years, %	14.0
12–14 years, %	10.6
≥15 years, %	21.9

^§^Abdominal obesity: waist circumference for men ≥97 cm, for women ≥89 cm; dyslipidemia: reported diagnosis of cholesterol problems; hypertension: reported diagnosis of high blood pressure/hypertension; prior cardiovascular event: reported heart disease/heart attack or stroke/TIA.

**Table 2:** Method of diagnosis for type 2 diabetes mellitus among SHIELD respondents.

Method of diagnosis	All respondents with type 2 diabetes mellitus *n* = 3 022	Type 2 diabetes mellitus selecting only one method of diagnosis^†^ *n* = 2 749
Routine screening, %	63.0	35.5
Testing after symptoms, %	49.4	20.3
Testing after treatment for another health problem, %	20.6	5.6

^†^percentages do not add to 100% since some respondents chose “other” or “home testing” as their method of diagnosis.

**Table 3:** Specialty of physician diagnosing type 2 diabetes mellitus among SHIELD respondents.

Specialty of physician making diagnosis of type 2 diabetes mellitus	Type 2 diabetes mellitus *n* = 2 660
Family doctor/general practitioner, %	88.3
Endocrinologist, %	4.4
Cardiologist, %	0.5
Neurologist, %	0.7
Other specialist, %	6.0
